# Organizational Socialization Among New Graduate Nurses: A Longitudinal Study Using Latent Profile and Transition Analyses

**DOI:** 10.1155/jonm/8841220

**Published:** 2026-10-15

**Authors:** Hee Jun Kim, Sun Hyoung Bae, Eun Ji Seo, Jin-Hee Park

**Affiliations:** ^1^ College of Nursing Eulji University Uijeongbu South Korea eulji.ac.kr; ^2^ College of Nursing Research Institute of Nursing Science Ajou University Suwon South Korea ajou.ac.kr

**Keywords:** adjustment outcomes, latent profile analysis, latent transition analysis, new graduate nurses, nurse retention, organizational socialization, turnover

## Abstract

**Aim:**

To identify the latent profiles of adjustment outcomes associated with organizational socialization among new graduate nurses at the end of the preceptorship period and approximately 10 months later, and to examine transitions in profile membership and to resignation.

**Background:**

Organizational socialization is critical for new graduate nurses to acquire the knowledge, skills, and professional identity required to function effectively within the organization. Most previous studies have employed a variable‐centered approach, which may obscure heterogeneity in adjustment patterns associated with organizational socialization.

**Methods:**

A longitudinal survey was conducted among 201 new graduate nurses employed at a tertiary hospital. Adjustment outcomes associated with organizational socialization were assessed using measures of nursing performance, work engagement, organizational commitment, and job embeddedness. Subgroups were identified at each point via latent profile analysis, and profile changes over time were examined using latent transition analysis.

**Results:**

Two profiles (less favorable and more favorable adjustment) were identified following the completion of the preceptorship period, whereas three profiles (less favorable, moderate, and more favorable adjustment) emerged approximately 10 months later. The profiles primarily reflected differences in the overall levels of the four adjustment outcomes, suggesting a low‐to‐high continuum rather than qualitatively distinct configurations. Nurses in the less favorable adjustment profile at the end of the preceptorship period had the highest estimated probability of subsequent resignation, whereas those in the more favorable adjustment profile had the highest estimated probability of remaining in the more favorable adjustment profile, indicating heterogeneity in adjustment profiles and their transitions over time.

**Conclusions:**

Identifying nurses with less favorable adjustment outcomes and providing structured and sustained support may facilitate adaptation and support retention. These findings should be interpreted in light of the use of adjustment outcomes to reflect organizational socialization and potential selection bias related to resignation during follow‐up.

**Implications for Nursing Management:**

The higher estimated probability of subsequent resignation among nurses in the less favorable adjustment profile highlights the importance of early identification of new graduate nurses with less favorable adjustment outcomes. The observed differences in adjustment profiles and their transitions suggest a need for support tailored to nurses’ adjustment needs and stages of transition. Integrating structured preceptorship and ongoing monitoring into workforce management may facilitate sustained adaptation and strengthen long‐term retention.

## 1. Introduction

The number of nursing students obtaining a registered nurse license in Korea has increased from approximately 15,400 in 2014 to 23,300 in 2022 [1]. While most graduates begin their professional careers as clinical nurses [2], new graduate nurses often exhibit reduced self‐confidence owing to their limited clinical knowledge and skills and experience difficulties in organizational socialization because of excessive workloads and insufficient coping abilities in new organizational environments [3]. According to a national survey report on nurse staffing published by the Hospital Nurses Association, the turnover rate among new graduate nurses was 34.1% in 2021, substantially higher than the overall nurse turnover rate of 14.2% [4]. Such a high turnover rate among new graduate nurses poses considerable challenges for workforce management in healthcare organizations, leading to repeated recruitment and retraining and contributing to workforce instability [5]. Furthermore, the failure to retain experienced nursing staff undermines the delivery of high‐quality patient care [6]. Accordingly, increasing attention has been directed toward organizational socialization in clinical settings because of its potential importance for supporting the successful adaptation and retention of new graduate nurses [7].

Organizational socialization refers to the integrative process through which individuals enter an organization and transition from complete outsiders to engaged and effective organizational members through learning and adaptation [8]. During this process, individuals develop and implement personal coping strategies to address workplace challenges while cultivating job‐related and psychological resources, including confidence and a sense of responsibility in role performance [9]. Importantly, organizational socialization is not confined to the initial period of employment and dynamically unfolds over time [8]. For new graduate nurses, the first year of employment represents a critical transition period during which they move beyond initial entry‐level roles and begin to assume substantive responsibilities as organizational members. During this period, an understanding of organizational culture deepens, and autonomy and accountability in professional practice become more firmly established [10].

Given the importance of continued adjustment during the first year of employment, organizational socialization approximately 1 year after entry may be associated with longer‐term career retention and job performance among nurses [11]. Thus, examining adjustment patterns associated with organizational socialization during this period is important. Furthermore, examining changes in these adjustment patterns during the first year of practice may provide insights for developing strategies to support the retention and professional growth of new graduate nurses [12].

Nonetheless, previous studies have primarily relied on a variable‐centered approach, which explains relationships among variables using a single summary estimate such as the mean. Although useful, the variable‐centered approach has limitations in capturing the multidimensional nature of human experiences and heterogeneity among individuals [13]. To address these limitations, increasing attention has been directed toward the person‐centered approach. One widely employed analytical method is latent profile analysis (LPA), which classifies individuals within a population into distinct latent subgroups according to similar characteristics, leading to the identification of heterogeneous subtypes [13]. This is particularly appropriate for examining heterogeneity in adjustment outcomes associated with organizational socialization among new graduate nurses, who may comprise subgroups with distinct characteristics and needs [12]. Latent transition analysis (LTA), a longitudinal extension of LPA, estimates how individuals transition between latent subgroups over time. As such, LTA serves as an important methodological tool for examining transitions in patterns of adjustment outcomes associated with organizational socialization among new graduate nurses [14].

In the present study, we aimed to identify latent profiles of adjustment outcomes associated with organizational socialization among new graduate nurses following the completion of the preceptorship period and approximately 10 months later and to examine transitions between these profiles over time.

## 2. Materials and Methods

### 2.1. Study Design

A longitudinal survey was conducted to identify latent profiles of adjustment outcomes associated with organizational socialization among new graduate nurses and to examine their transitions over time. Repeated measurements were performed upon completion of the 8‐week preceptorship period (T1) and approximately 10 months thereafter (T2). Profiles were identified at each time point using LPA, and transitions from the T1 profiles to the T2 profiles or resignation were examined using LTA.

### 2.2. Theoretical Framework

The theoretical framework of this study is grounded in Bauer et al.’s organizational socialization model [8], which explains the socialization process through which newcomers transition from organizational outsiders to insiders through learning and adaptation. This model identifies key socialization attributes (e.g., role clarity, self‐efficacy, and acceptance of organizational values) that subsequently lead to important adjustment outcomes, including performance, job satisfaction, organizational commitment, and intention to remain. These outcomes reflect the extent to which newcomers have successfully adjusted and integrated into the organization and thus provide observable manifestations of adjustment associated with organizational socialization [8]. Within this framework, organizational socialization is conceptualized not merely as an initial adjustment phase but as a dynamic process influencing the job performance and long‐term integration of individuals in an organization [8, 12].

Guided by this theoretical perspective, nursing performance, work engagement, organizational commitment, and job embeddedness were selected as indicators of adjustment outcomes associated with organizational socialization in the nursing context [11, 15]. Work engagement was included to reflect an active and positive psychological state toward work, whereas job embeddedness was included to capture broader relational and organizational attachment beyond the intention to remain. Accordingly, the latent profiles identified in this study should be interpreted as multidimensional patterns of adjustment outcomes associated with organizational socialization rather than as direct measures of the organizational socialization process itself [8]. Latent profiles of these adjustment outcomes and their transitions over time were examined based on this operationalization.

### 2.3. Study Participants

This study included new graduate nurses who were newly employed at a tertiary hospital in Gyeonggi Province, Korea, from June 2023 to December 2024. Nurses were eligible for inclusion if they were new graduate nurses newly employed at the study hospital, provided direct patient care, and had no previous work experience at other hospitals. Nurses who were assigned to departments that did not involve direct patient care or were employed in temporary or contract‐based positions were excluded.

Initially, 204 participants were recruited. Of these, one participant who did not consent to longitudinal follow‐up and therefore did not provide contact information, one participant who remained employed but did not respond to the T2 follow‐up survey, and one participant with missing data on at least one of the adjustment outcome indicators (nursing performance, work engagement, organizational commitment, or job embeddedness) were excluded. Thus, data from 201 participants were included in the final analysis. Although no definitive sample size standard has been established for LTA, simulation research has shown accurate model selection with sample sizes of 200 or greater under the conditions examined [16], supporting the adequacy of the present sample. All 201 participants had complete data for the adjustment outcome indicators at T1. At T2, 146 participants remained employed and completed the adjustment outcome measures, whereas 55 had resigned during the follow‐up period. Because adjustment outcome measures were unavailable after resignation, the latent profiles at T2 were derived from the 146 participants who remained employed.

### 2.4. Measures

#### 2.4.1. Sociodemographic Variables

General characteristics included the participants’ sex, current work department, assignment to their preferred department, and level of salary satisfaction.

#### 2.4.2. Adjustment Outcomes Associated With Organizational Socialization

Nursing performance, work engagement, organizational commitment, and job embeddedness served as the four indicators for the LPAs. Together, these indicators captured multidimensional adjustment outcomes associated with organizational socialization, rather than the socialization process itself.

##### 2.4.2.1. Nursing Performance

Nursing performance was assessed using the Nursing Performance Scale developed by Ko et al. [17], a 17‐item instrument with four subdomains, namely, nursing competency (seven items), nursing performance attitude (four items), improvement in nursing practice (three items), and application of the nursing process (three items). Each item is rated on a five‐point Likert scale ranging from 1 (“Strongly Disagree”) to 5 (“Strongly Agree”), with higher scores indicating better nursing performance. Cronbach’s alpha was 0.84 in the original study by Ko et al. [17] and 0.91 in the present study, indicating high internal consistency.

##### 2.4.2.2. Work Engagement

Work engagement was evaluated using the nine‐item Utrecht Work Engagement Scale (UWES‐9), originally developed by Schaufeli et al. [18] and translated into Korean by Kim et al. [19]. The UWES‐9 consists of three subdimensions: vigor, dedication, and absorption. Each item is rated on a seven‐point Likert scale ranging from 0 (“Never”) to 6 (“Always”), with higher scores indicating greater work engagement. Cronbach’s alpha ranged from 0.85 to 0.92 in previous studies and was 0.94 in the present study, indicating excellent internal consistency.

##### 2.4.2.3. Organizational Commitment

Organizational commitment was assessed using the 15‐item Organizational Commitment Scale, originally developed by Mowday et al. [20] and translated and revised for Korean nurses by Lee [21]. Each item is rated on a seven‐point Likert scale ranging from 1 (“Strongly Disagree”) to 7 (“Strongly Agree”), with higher scores indicating greater organizational commitment. Cronbach’s alpha was 0.91 in Lee’s previous study involving nurses [21] and 0.93 in the present study, indicating excellent internal consistency.

##### 2.4.2.4. Job Embeddedness

Job embeddedness was evaluated using the instrument originally developed by Mitchell et al. [22], subsequently adapted for nurses by Reitz [23], and translated into Korean and validated by Choi and Lee [24]. The scale consists of 17 items across four subdomains: organizational fit (eight items), organizational sacrifice (four items), community fit (three items), and work team (two items). Each item is rated on a five‐point Likert scale ranging from 1 (“Strongly Disagree”) to 5 (“Strongly Agree”), with higher scores indicating greater job embeddedness. Cronbach’s alpha ranged from 0.84 to 0.87 in previous studies [22–24] and was 0.87 in the present study, indicating acceptable internal consistency.

### 2.5. Data Collection

Data were collected after the participants received a full explanation of the study’s objectives from the researcher or a trained research assistant and provided written informed consent. All questionnaires were completed in person by the participants. All new graduate nurses received the same 3‐day institutionwide orientation, followed by an 8‐week preceptorship program. The preceptorship program included basic clinical nursing practice, patient safety, communication, and handover skills and was standardized across clinical units using a single institutional preceptorship guideline developed by the hospital. Thus, the overall structure, content, and duration of the orientation and preceptorship program were consistent across participants.

Data were collected at two time points. The first assessment (T1) was conducted upon completion of the 8‐week preceptorship period, when participants transitioned to independent nursing practice, and the second assessment (T2) was conducted approximately 10 months after T1. At both time points, participants completed standardized self‐administered questionnaires in a one‐on‐one, face‐to‐face setting with either the researcher or a trained research assistant. The researcher or research assistant provided procedural guidance but did not administer or record questionnaire responses on behalf of the participants. The timing of the assessments and data collection procedures were consistently applied across participants.

### 2.6. Data Analysis

Latent profiles based on the four adjustment outcomes associated with organizational socialization were identified at each time point using LPA. LPA at T1 was conducted using data from all 201 participants, whereas LPA at T2 was conducted using data from the 146 participants who remained employed and completed the four adjustment outcome measures. LTA was subsequently conducted to estimate transitions from the T1 latent profiles to the T2 profiles or resignation. Resignation during the follow‐up period was treated as a separate state in the LTA rather than as missing follow‐up data, allowing the probability of transition from each T1 profile to resignation to be estimated.

Multiple model fit indices, including the Bayesian information criterion (BIC), sample size–adjusted BIC (SABIC), Lo–Mendell–Rubin likelihood ratio test (LMR‐LRT), bootstrap likelihood ratio test (BLRT), and entropy, were considered to determine the optimal number of latent profiles at each time point. Model fit was primarily evaluated using the BIC and SABIC, with lower values indicating better model fit. Model comparisons were further performed using the LMR‐LRT and BLRT by comparing the likelihood of the *k*‐class model with that of the *k* − 1‐class model. A nonsignificant *p* value denoted that the more parsimonious *k* − 1‐class model was preferable. Classification accuracy was assessed using entropy (range: 0–1), with values closer to 1 indicating greater precision in profile classification.

Additionally, to assess potential selection bias associated with resignation during follow‐up, T1 characteristics and adjustment outcome indicators were compared between nurses who remained employed and those who resigned using independent‐samples *t* tests and chi‐square tests, as appropriate. Statistical analyses were performed using Jamovi Version 2.3.28 and Mplus Version 8.8.

### 2.7. Ethical Considerations

This study was conducted in accordance with the ethical principles outlined in the Declaration of Helsinki and was approved by the Institutional Review Board of the researchers’ affiliated institution (Approval No.: AJOUIRB‐SB‐2023‐259). All participants were provided with detailed information about the study’s purpose, data collection procedures, and ethical considerations. Participation was voluntary, and written informed consent was obtained from all participants prior to data collection.

## 3. Results

### 3.1. General Characteristics of the Participants

Following the completion of the preceptorship period, 201 new graduate nurses participated at T1. Of these, 146 remained employed and completed the T2 assessment approximately 10 months later, resulting in a follow‐up rate of 72.6%, whereas 55 nurses resigned during the follow‐up period. Resignation status was verified using institutional human resource records. The general characteristics of the participants are summarized in Table 1. At T1, 91.5% of participants were female, 56.2% were assigned to general wards, 67.7% were assigned to their preferred departments, and > 90% reported being either “neutral” or “satisfied” with their salary.

**TABLE 1:** General characteristics of participants at the completion of the preceptorship period and approximately 10 months later.

Variable	Postpreceptorship (*N* = 201)	10‐month follow‐up (*N* = 146)
	Mean ± SD or *n* (%)	
Age (years)	25.48 ± 1.05	26.44 ± 1.02
Sex		
Female	184 (91.5)	131 (89.7)
Male	17 (8.5)	15 (10.3)
Department		
Ward	113 (56.2)	78 (53.4)
ICU	65 (32.3)	48 (32.9)
Others	23 (11.5)	20 (13.7)
Placement in desired department		
Yes	136 (67.7)	106 (72.6)
No	65 (32.3)	40 (27.4)
Salary satisfaction		
Dissatisfied	12 (6.0)	5 (3.4)
Neutral	97 (48.2)	65 (44.5)
Satisfied	92 (45.8)	76 (52.1)

*Note: n*, number; *N*, sample size.

Abbreviations: SD, standard deviation; ICU, intensive care unit.

T1 characteristics and adjustment outcome indicators were compared between the 146 nurses who remained employed and the 55 nurses who resigned during follow‐up. Nurses who resigned were less likely to have been assigned to their preferred department (*p* = 0.015) and had lower salary satisfaction (*p* = 0.003), nursing performance (*p* < 0.001), organizational commitment (*p* = 0.001), and job embeddedness (*p* < 0.001) at T1. No significant differences were found in age, sex, clinical department, or work engagement. Detailed results are presented in Supporting Table 1.

### 3.2. Model Fit Indices for the LPAs

Model fit indices for the LPA at T1 and T2 are presented in Table 2. At T1, following the completion of the preceptorship period, the BIC and SABIC decreased as the number of profiles increased. The LMR‐LRT and BLRT were significant for the two‐ and four‐profile models, whereas the LMR‐LRT was not significant for the three‐profile model (*p* = 0.365), indicating that adding a third profile did not significantly improve model fit over the two‐profile solution. Although the four‐profile solution showed lower BIC and SABIC values, it included two comparatively small profiles (10.4% and 8.1%) and did not provide sufficiently distinct patterns with clear substantive or practical relevance to justify the additional model complexity. In contrast, the two‐profile solution yielded two adequately sized and readily interpretable profiles (54.0% and 46.0%) with acceptable classification accuracy (entropy = 0.805). Therefore, considering statistical fit, profile size, parsimony, interpretability, and substantive relevance, the two‐profile solution was selected as the optimal model.

**TABLE 2:** Model fit indices for latent profiles of adjustment outcomes associated with organizational socialization.

Time	Model	BIC	SABIC	LMR‐LRT	BLRT	Entropy	Classification proportion				
							1	2	3	4	5
Postpreceptorship	2	1372.65	1331.46	0.0018	< 0.001	0.805	0.540	0.460			
	3	1295.85	1238.82	0.3652	< 0.001	0.829	0.384	0.500	0.116		
	4	1235.56	1162.69	0.0105	< 0.001	0.870	0.104	0.438	0.081	0.377	
	5	1229.05	1140.34	0.1699	< 0.001	0.848	0.105	0.274	0.333	0.231	0.057

10‐month follow‐up	2	834.67	793.53	0.0085	< 0.001	0.785	0.491	0.509			
	3	788.48	731.52	0.0001	< 0.001	0.839	0.257	0.456	0.287		
	4	791.31	718.53	0.2876	< 0.001	0.866	0.252	0.290	0.426	0.032	
	5	806.04	717.43	0.3670	0.3750	0.845	0.142	0.119	0.414	0.288	0.037

*Note:* For the selected models, both the LMR‐LRT and BLRT were statistically significant (*p* < 0.05). SABIC, sample size–adjusted Bayesian information criterion.

Abbreviations: BIC, Bayesian information criterion; BLRT, bootstrap likelihood ratio test; LMR‐LRT, Lo–Mendell–Rubin likelihood ratio test.

At T2, approximately 10 months after T1, the three‐profile model showed the lowest BIC value. The LMR‐LRT and BLRT were significant for the two‐ and three‐profile solutions. Entropy values remained > 0.80, indicating stable classification. Based on overall model fit and theoretical interpretability, the three‐profile solution was considered the most appropriate. The proportions of participants were 25.7%, 45.6%, and 28.7% in the three profiles.

### 3.3. Latent Profiles at the Completion of Preceptorship and Follow‐Up

The latent profiles based on the four organizational socialization‐related adjustment outcomes at T1 and T2 are presented in Table 3. At T1, the LPA of all 201 participants identified a two‐profile solution. Profile 1, which comprised 54.0% of the participants, was characterized by low levels of nursing performance, work engagement, organizational commitment, and job embeddedness; this group was labeled the “less favorable adjustment profile.” Profile 2, which comprised 46.0% of the participants, exhibited high levels across all four variables; this group was labeled the “more favorable adjustment profile.”

**TABLE 3:** Latent profiles of adjustment outcomes associated with organizational socialization.

Time	Variables	Total (*N* = 201)	Less favorable adjustment profile (*N* = 108)	More favorable adjustment profile (*N* = 93)	
		M ± SD	M ± SE	M ± SE	
Postpreceptorship	Nursing performance	3.49 ± 0.49	3.30 ± 0.05	3.73 ± 0.06	
	Work engagement	3.03 ± 0.99	2.45 ± 0.12	3.72 ± 0.12	
	Organizational commitment	4.98 ± 0.79	4.41 ± 0.09	5.66 ± 0.11	
	Job embeddedness	3.41 ± 0.44	3.13 ± 0.05	3.73 ± 0.06	

**Time**	**Variables**	**Total (*N* = 146)**	**Less favorable adjustment profile (*N* = 37)**	**Moderate adjustment profile (*N* = 69)**	**More favorable adjustment profile (*N* = 40)**
		**M ± SD**	**M ± SE**	**M ± SE**	**M ± SE**

10‐month follow‐up	Nursing performance	3.77 ± 0.44	3.52 ± 0.06	3.69 ± 0.05	4.13 ± 0.05
	Work engagement	3.05 ± 0.80	2.32 ± 0.12	3.04 ± 0.08	3.74 ± 0.11
	Organizational commitment	4.89 ± 0.66	4.15 ± 0.06	4.86 ± 0.07	5.61 ± 0.08
	Job embeddedness	3.48 ± 0.38	2.99 ± 0.04	3.50 ± 0.04	3.88 ± 0.04

*Note: N*, sample size; M, mean.

Abbreviations: SD, standard deviation; SE, standard error.

At T2, approximately 10 months after T1, 55 of the original 201 participants had resigned, leaving data from 146 nurses for analysis. The LPA of these 146 participants identified a three‐profile solution. Profile 1 (25.7%) was characterized by low levels across all four variables and was labeled the “less favorable adjustment profile.” Profile 2 (45.6%) showed moderate levels across all variables and was labeled the “moderate adjustment profile.” Profile 3 (28.7%) exhibited high levels across all variables and was labeled the “more favorable adjustment profile.”

### 3.4. Transition Probabilities Between Latent Profiles

Transition probabilities from T1 to T2 are shown in Table 4. For nurses in the less favorable adjustment profile at T1, the estimated probability of transition to resignation was 38.4%. The estimated probabilities of remaining in the less favorable adjustment profile and transitioning to the moderate adjustment profile and the more favorable adjustment profile were 28.9%, 27.2%, and 5.5%, respectively. For nurses in the more favorable adjustment profile at T1, the estimated probability of remaining in the more favorable adjustment profile at T2 was 43.2%. The estimated probabilities of transitioning to the moderate adjustment profile, resignation, and the less favorable adjustment profile were 32.7%, 14.5%, and 9.6%, respectively.

**TABLE 4:** Transition probabilities among adjustment profiles associated with organizational socialization and resignation.

Time	Profiles	10‐month follow‐up *n* (estimated transition probability, %)			
		Less favorable adjustment profile (*N* = 40)	Moderate adjustment profile (*N* = 60)	More favorable adjustment profile (*N* = 46)	Resignation (*N* = 55)
Post‐preceptorship	Less favorable adjustment profile (*N* = 108)	31 (28.9)	30 (27.2)	6 (5.5)	41 (38.4)
	More favorable adjustment profile (*N* = 93)	9 (9.6)	30 (32.7)	40 (43.2)	14 (14.5)

*Note:* Values are presented as *n* (estimated transition probability, %). Transition probabilities were estimated using latent transition analysis and therefore may not correspond exactly to percentages calculated directly from the observed counts. Class counts shown in Table 4 are based on the latent transition analysis classification and may therefore differ from the LPA class counts reported in Table 3. *n*, number; *N*, sample size.

## 4. Discussion

In this study, we identified patterns of adjustment associated with organizational socialization among new graduate nurses and examined their transitions over time. At T1, two profiles were identified: the less favorable adjustment profile (54.0%) and the more favorable adjustment profile (46.0%), with the latter showing uniformly higher levels across all four variables. These profiles primarily reflected differences in the overall level of adjustment outcomes associated with organizational socialization rather than qualitatively distinct configurations, suggesting a low‐to‐high continuum of adjustment. These findings suggest that a substantial proportion of new graduate nurses transition to independent practice with relatively low levels of adjustment, potentially reflecting challenges associated with early clinical transition, including limited practical competence and the theory–practice gap [25].

In this study, the first assessment was conducted upon the completion of the preceptorship period, a critical transition point at which new graduate nurses begin to perform nursing duties independently [10]. In Korean healthcare institutions, various transition programs, including structured preceptorships, are implemented to support clinical adaptation and skill development among newly hired nurses. Although the duration and content of these programs vary across hospitals and departments, they generally provide practice‐based instruction and supervision by experienced nurses for a defined period [26].

During the preceptorship period, new graduate nurses gradually perform nursing tasks under the guidance and support of experienced nurses. However, once independent practice begins, they assume full responsibility for patient care and are expected to make timely and appropriate clinical decisions in unexpected situations [27]. This increased responsibility may contribute to psychological strain [28]. Evidence from demanding clinical settings has also shown that compassion fatigue and moral distress may be interrelated [29], suggesting that emotional strain may further complicate nurses’ psychological adaptation to practice. For new graduate nurses, such psychological strain may make adaptation to independent practice more challenging and, in turn, hinder effective organizational socialization [15]. Accordingly, organizational socialization programs should incorporate both structured, practice‐oriented educational interventions aimed at reducing the theory–practice gap and ongoing psychological support. Continuous organizational support, including phased task allocation after the transition to independent practice, regular performance evaluation and feedback, support from nurse educators, and regular psychological check‐ins and opportunities for debriefing, should therefore be maintained beyond the preceptorship period.

At T2, three level‐based profiles of adjustment outcomes associated with organizational socialization were identified among the nurses who remained employed, namely, the less favorable adjustment profile (25.7%), the moderate adjustment profile (45.6%), and the more favorable adjustment profile (28.7%). These T2 profiles should therefore be interpreted in light of attrition and the potential for selection bias. The presence of these profiles indicates that adjustment outcomes varied among nurses approximately 10 months after the completion of the preceptorship period. This variation suggests that nurses at a similar stage of employment may have different support needs. Accordingly, support strategies should be tailored to nurses’ adjustment profiles rather than based solely on time since entry into practice.

The transition analysis further illustrates changes in adjustment profile membership among new graduate nurses between the two assessments. For nurses in the less favorable adjustment profile at T1, the estimated probabilities of remaining in the same profile and transitioning to the moderate adjustment and the more favorable adjustment profiles at T2 were 28.9%, 27.2%, and 5.5%, respectively. For nurses in the more favorable adjustment profile at T1, the estimated probability of remaining in the same profile at T2 was 43.2%, whereas the estimated probabilities of transitioning to the moderate adjustment profile and the less favorable adjustment profile were 32.7% and 9.6%, respectively. These findings are consistent with the results of a previous study examining organizational adaptation trajectories [12], which reported stratified high, moderate, and low adaptation patterns over time and showed that individuals in higher‐level trajectories tended to maintain their level, whereas those in lower‐level trajectories often persisted without significant improvement [12]. Collectively, these results underscore the importance of identifying new graduate nurses with less favorable adjustment outcomes early in employment. Targeted and structured educational and support interventions may help address their adjustment needs and support long‐term retention.

In this study, the estimated probability of transition to resignation by T2 was 38.4% for nurses in the less favorable adjustment profile at T1 and 14.5% for those in the more favorable adjustment profile, indicating differences in the estimated probability of subsequent resignation across initial adjustment profiles. Recent person‐centered research among novice nurses has also identified distinct safety‐attitude profiles and demonstrated that these profiles differentially buffered the relationship between occupational identity and turnover intention [30]. Although safety attitudes and organizational socialization represent distinct aspects of novice nurses’ work experiences, these findings similarly underscore the importance of recognizing individual differences in adaptation and identifying nurses who may require additional support. These findings also imply that the preceptorship period should not merely be regarded as a phase of technical skill training; rather, it functions as a critical mechanism of organizational socialization that facilitates newcomers’ adaptation as organizational members and fosters a sense of belonging [31]. Indeed, education provided during the overlapping work period extends beyond improving clinical competence and encompasses multidimensional socialization processes, including the development of role clarity, strengthening of self‐efficacy, internalization of organizational values, formation of collegial relationships, and acquisition of effective information‐seeking strategies [12, 32].

Preceptors, in particular, function not only as clinical instructors but also as providers of psychological support and mediators of organizational culture. By contributing to emotional stability and fostering a sense of shared understanding, they play a pivotal role in strengthening new graduate nurses’ sense of belonging within the organization [31]. Effective preceptorship may also facilitate new graduate nurses’ active help‐seeking by providing a supportive environment in which they can recognize difficulties, ask questions, and seek guidance when needed. Help‐seeking has been conceptualized as an active process involving the recognition of the need for support, decisions to seek assistance, and timely communication of that need [33]. Although this conceptualization was developed in a patient context, it highlights the potential importance of timely help‐seeking during new graduate nurses’ transition to practice. Preceptors should therefore foster an environment in which asking questions and seeking assistance are regarded as appropriate professional behaviors rather than as signs of insufficient competence, thereby enabling new graduate nurses to access timely guidance and feedback. Thus, preceptorship can support clinical learning and active engagement with available support, which may facilitate adaptation and organizational socialization.

Furthermore, effective preceptorship has been associated with greater job satisfaction and organizational commitment and lower turnover intention among new graduate nurses [34, 35]. Evidence from other clinical contexts has also shown that structured nurse‐led education and support can improve clinical and behavioral outcomes compared with usual care [36], further supporting the broader value of systematically structured nurse‐led support. Taken together, these findings suggest that early stage training programs should be designed with sufficient duration and flexibility to accommodate individual differences in competence and the pace of adaptation. Educational content should extend beyond task‐oriented instruction to include strategies that facilitate help‐seeking, relationship building, and active participation in the socialization process. Additionally, healthcare organizations should ensure adequate staffing for educational roles and implement systematic support strategies to strengthen preceptors’ teaching competence and improve the overall quality of preceptorship programs [37].

This study identified heterogeneous profiles of adjustment outcomes associated with organizational socialization among new graduate nurses and transitions between these profiles over time. Notably, the T1 profiles showed different probabilities of transition to the T2 adjustment profiles and to resignation. These findings suggest that management strategies for new graduate nurses should not be uniformly applied. Instead, strengthening early stage education and implementing tailored stage‐specific support strategies (e.g., monthly peer support groups, structured check‐ins with nurse educators, resilience training, continued clinical coaching after transition to independent practice, and individualized mentoring) are essential. Furthermore, individualized interventions based on profile‐specific characteristics may help support organizational adjustment and long‐term retention among new graduate nurses.

Despite these contributions, this study has some limitations. First, the identified profiles should be interpreted as multidimensional patterns of adjustment outcomes associated with organizational socialization rather than as direct empirical representations of the organizational socialization process itself. This interpretation reflects the use of nursing performance, work engagement, organizational commitment, and job embeddedness as indicators of adjustment outcomes associated with organizational socialization. Second, the sample was drawn from new graduate nurses employed at a single tertiary hospital in Gyeonggi Province, Korea, which may limit the generalizability of the findings to other regions or healthcare settings. Organizational culture, staffing conditions, the quality and consistency of preceptorship, and regional labor market conditions may influence organizational socialization and resignation and may therefore affect the observed transition probabilities. Future multisite studies should directly assess these contextual factors and examine whether the identified profile structures and transition probabilities are replicated across institutions and regions.

Third, the data were based on self‐reported questionnaires, raising the possibility of social desirability bias. In particular, new graduate nurses may have reported their nursing performance or organizational commitment more favorably in an effort to appear competent or well adjusted. Future studies should combine self‐reported data with supervisor assessments, objective performance indicators, and administrative records. Fourth, potential selection bias associated with resignation during follow‐up should be considered. Although resignation was included as a separate state in the transition analysis, adjustment outcomes were unavailable for the 55 nurses who resigned; therefore, the T2 profiles were derived only from the 146 nurses who remained employed. Nurses who resigned had lower T1 nursing performance, organizational commitment, and job embeddedness scores (Supporting Table 1), suggesting that nurses with less favorable initial adjustment outcomes may be underrepresented in the T2 profiles. Treating resignation as a separate state captures transitions to resignation but does not rule out potential selection bias in the T2 profiles. Accordingly, these profiles may not fully represent the adjustment patterns of the original cohort. Fifth, this study relied on two measurement points, namely, upon the completion of the preceptorship period and approximately 10 months later, which may not fully capture intermediate changes or more nuanced longitudinal patterns in adjustment outcomes associated with organizational socialization. Finally, in the LPA and LTA, relatively small subgroup sizes may have contributed to potential instability in parameter estimates. Future studies involving larger samples and multiple institutions should be conducted to validate and extend the present findings.

## 5. Conclusions

This study longitudinally identified patterns of adjustment outcomes associated with organizational socialization among new graduate nurses following the completion of the preceptorship period and approximately 10 months later, as well as transitions across adjustment profiles and to resignation. Two profiles (less favorable and more favorable adjustment profiles) were identified at the first assessment, whereas three profiles (less favorable, moderate, and more favorable adjustment profiles) emerged at follow‐up, indicating heterogeneous patterns of adjustment over time. Importantly, nurses in the profile characterized by lower adjustment outcome scores at the first assessment had a higher estimated probability of subsequent resignation.

These findings underscore the importance of the early identification of nurses experiencing less favorable adjustment outcomes and highlight the need for intensive and structured support during the initial adaptation period, including but not limited to preceptorship programs. Targeted early interventions may help promote organizational integration and support the retention of new graduate nurses.

## Author Contributions

Hee Jun Kim: conceptualization, methodology, data curation, formal analysis, and writing–original draft preparation.

Sun Hyoung Bae: methodology, writing–original draft preparation, and writing–review and editing.

Eun Ji Seo: methodology, writing–original draft preparation, and writing–review and editing.

Jin‐Hee Park: conceptualization, supervision, project administration, writing–original draft preparation, and writing–review and editing.

## Funding

This paper was supported by Eulji University in 2026(EJRG‐26‐14).

## Disclosure

All authors have read and approved the final manuscript.

## Ethics Statement

This study was conducted in accordance with the principles of the Declaration of Helsinki and was approved by the Institutional Review Board of Ajou University (Approval No.: AJOUIRB‐SB‐2023‐259).

## Consent

Written informed consent was obtained from all participants prior to data collection. Participation was voluntary, and the participants were informed that they could withdraw at any time without penalty.

## Conflicts of Interest

The authors declare no conflicts of interest.

## Supporting Information

Additional supporting information can be found online in the Supporting Information section.

## Supporting information


**Supporting Information** Supporting Table 1 presents the comparison of baseline characteristics and organizational socialization‐related adjustment outcomes between nurses who remained employed and those who resigned.

## Data Availability

The data are not publicly available due to ethical restrictions but are available from the corresponding author upon reasonable request.
