# Supplementary material for: Organizational Socialization Among New Graduate Nurses: A Longitudinal Study Using Latent Profile and Transition Analyses
Source: J Nurs Manag. 2026 Oct 15;2026:8841220. doi: 10.1155/jonm/8841220 (PMC13670136; doi:10.1155/jonm/8841220)
Supplement: Supplementary file 1 [file JONM-2026-8841220-s001.docx]

**Supplementary Table 1** | Comparison of Baseline Characteristics and Adjustment Outcomes Associated with Organizational Socialization Between Nurses Who Remained Employed and Those Who Resigned

| **Variables** | **Total**  **(*N* = 201)** | **Employed nurses**  **(*N* = 146)** | **Resigned nurses**  **(*N* = 55)** | ***t* or χ² *(p)*** | |  |
| --- | --- | --- | --- | --- | --- | --- |
|  | **Mean ± SD or n (%)** | | |  |  |  |
| Age, years | 25.48 ± 1.05 | 25.44 ± 1.02 | 25.58 ± 1.15 | -0.86 (0.391) | |  |
| Sex |  |  |  |  | |  |
| Female | 184 (91.5) | 131 (71.2) | 53 (28.8) | (0.163†) | |  |
| Male | 17 (8.5) | 15 (88.2) | 2 (11.8) |  |  |  |
| Department |  |  |  |  | |  |
| Ward | 113 (56.2) | 78 (69.0) | 35 (31.0) | 3.16 (0.206) | |  |
| ICU | 65 (32.3) | 48 (73.8) | 17 (26.2) |  |  |  |
| Others | 23 (11.5) | 20 (87.0) | 3 (13.0) |  |  |  |
| Placement in desired department |  |  |  |  |  |  |
| Yes | 136 (67.7) | 106 (77.9) | 30 (22.1) | 5.95 (0.015) | | |
| No | 65 (32.3) | 40 (61.5) | 25 (38.5) |  |  |  |
| Salary satisfaction |  |  |  |  | | |
| Dissatisfied | 12 (6.0) | 5 (41.7) | 7 (58.3) | 11.94 (0.003) | | |
| Neutral | 97 (48.2) | 65 (67.0) | 32 (33.0) |  | | |
| Satisfied | 92 (45.8) | 76 (82.6) | 16 (17.4) |  | | |
| Nursing performance | 3.49 ± 0.49 | 3.57 ± 0.44 | 3.30 ± 0.54 | 3.56 (<0.001) | | |
| Work engagement | 3.03 ± 0.99 | 3.09 ± 0.98 | 2.89 ± 1.02 | 1.27 (0.204) | | |
| Organizational commitment | 4.98 ± 0.79 | 5.09 ± 0.77 | 4.69 ± 0.78 | 3.25 (0.001) | | |
| Job embeddedness | 3.41 ± 0.44 | 3.48 ± 0.42 | 3.21 ± 0.44 | 3.93 (<0.001) | | |

*Note:* † Fisher’s exact test

Abbreviations: n, number; N, sample size; SD, standard deviation; ICU, intensive care unit
